# A Case of Complete Resolution of Repeated Syncope Attacks After a Right-Sided Carotid Endarterectomy

**DOI:** 10.7759/cureus.63567

**Published:** 2024-07-01

**Authors:** Shuki Mizukami, Akihito Hashiguchi, Kensuke Sasaki, Koichi Moroki, Hajime Tokuda

**Affiliations:** 1 Neurological Surgery, Tokuda Neurosurgical Hospital, Kanoya, JPN

**Keywords:** autonomic nerve system, insular cortex, recurrent syncope, carotid endarterectomy (cea), carotid artery stenosis

## Abstract

Syncope is a common clinical entity with variable presentations and often an elusive causal mechanism, even after extensive evaluation. In any case, global cerebral hypoperfusion, resulting from the inability of the circulatory system to maintain blood pressure (BP) at the level necessary to supply blood to the brain efficiently, is the final pathway for syncope. Steno-occlusive carotid artery disease, even if bilateral, does not usually cause syncope. However, the patient presented here had repeated syncope attacks and underwent a thorough examination for suspected cardiac disease, but no abnormality was found. Since there was severe stenosis in the right unilateral internal carotid artery (ICA), but no stenosis in the left ICA or vertebrobasilar artery (VBA), and transient left mild hemiparesis associated with syncope, carotid revascularization surgery for the right ICA was performed, and the repeated syncope attacks completely disappeared after the surgery. The patient's condition improved markedly, and no further episodes of syncope have been reported. We report the relationship between carotid artery stenosis and syncope and discuss its pathomechanism.

## Introduction

Even if bilateral, steno-occlusive carotid artery disease rarely causes syncope. A decrease in systemic blood pressure (BP) induced by antihypertensive drugs or dehydration causes syncope when cerebral blood flow (CBF) in the global cerebral hemispheres is reduced and CBF to the mesencephalon and diencephalon, including the reticular formation and its ascending tract, which control consciousness, cannot be maintained [1,2]. On the other hand, brain lateralization concerning autonomic function has been extensively studied, with reports implicating various regions. However, many reports point to the involvement of the insular cortex in particular. Although definitive conclusions in humans have not yet been reached, many reports support the possibility that the right insular cortex is involved in sympathetic activation and the left insular cortex in parasympathetic activation [3-7]. This understanding may have important implications for our understanding of autonomic function. For example, we report here the case of an older woman whose recurrent syncope attacks completely resolved after a carotid endarterectomy (CEA) for right-sided unilateral internal carotid artery (ICA) stenosis. This fact suggests that insufficient right insular CBF associated with severe stenosis of the right ICA caused a relative decrease in sympathetic activity, leading to recurrent syncope attacks.

## Case presentation

**Figure 1:**
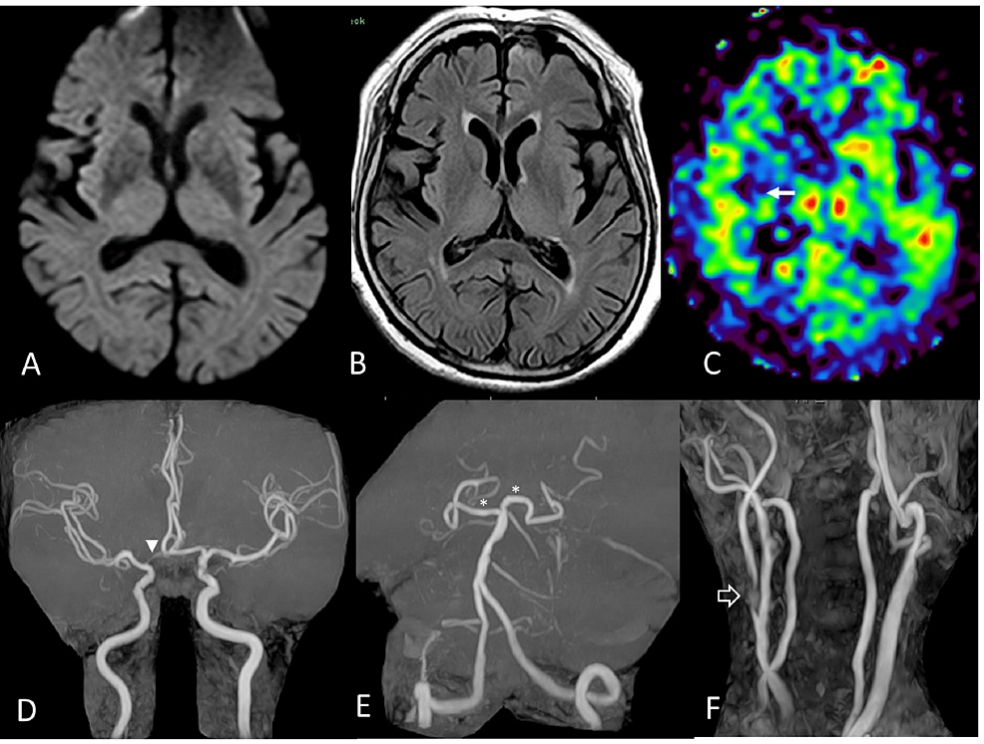
Preoperative magnetic resonance imaging (MRI) (A) MR diffusion-weighted imaging shows no acute infarction with the hyperintense signal. A metallic artifact of unknown origin is present in the left frontal region. (B) Fluid-attenuated inversion recovery (FLAIR) MRI shows no abnormalities around the right insular cortex. (C) Arterial-spin-labelling MRI shows decreased cerebral blood flow around the right insular cortex (arrow) compared to the left side. (D) There is no marked stenosis of the main intracranial artery. However, the A1 portion (arrow head) of the right anterior cerebral artery is narrow and nondominant. (E) The proximal portions (asterisk) of the bilateral posterior cerebral arteries are clearly demonstrated due to the posterior communicating arteries of the adult type. (F) Cervical MR angiography (MRA) shows severe stenosis with hyperintense unstable plaque at the origin of the right internal carotid artery (white arrow). There is no significant stenosis in the left internal carotid artery.

An older woman in her late 80s with a history of hypertension, who had not had any episodes of loss of consciousness in her youth, was referred to a cardiologist at another hospital to search for the cause of repeated syncope attacks that had been occurring for about a year. All syncope attacks occurred sitting or standing without clinical seizure activity and spontaneously resolved within minutes. She regained consciousness and was fully oriented without focal symptoms or signs. Her usual systolic BP was around 150 mmHg, but when she lost consciousness, her systolic BP was around 110 mmHg, and her heart rate was in the 70s. Her cardiac function was good, no bradyarrhythmia was observed, and the cause of the syncope could not be identified. However, a carotid ultrasound revealed a right ICA peak velocity of 2.32m/s, interpreted as right ICA stenosis of >70% by the North American Symptomatic Carotid Endarterectomy Trial (NASCET) method. Since she was also suspected to have left mild hemiparesis during syncope, and since severe stenosis of the right ICA was suspected as the cause of the syncope attack, the patient was referred to our hospital. She had normal neurological findings, including cognitive function. However, even during the examination, we could see syncope attacks induced by sitting for several minutes, which spontaneously resolved when she was supine. Although the plan was to carefully consider the indication for carotid surgery after evaluating CBF while conservatively monitoring the patient with adequate fluid replacement and antiplatelet medications, the frequent syncope attacks induced by the elevation of the head position led to a policy of early carotid artery revascularization. Preoperative magnetic resonance imaging (MRI) (Figure 1) provided precise, detailed results showing no fresh cerebral infarction.

**Figure 2:**
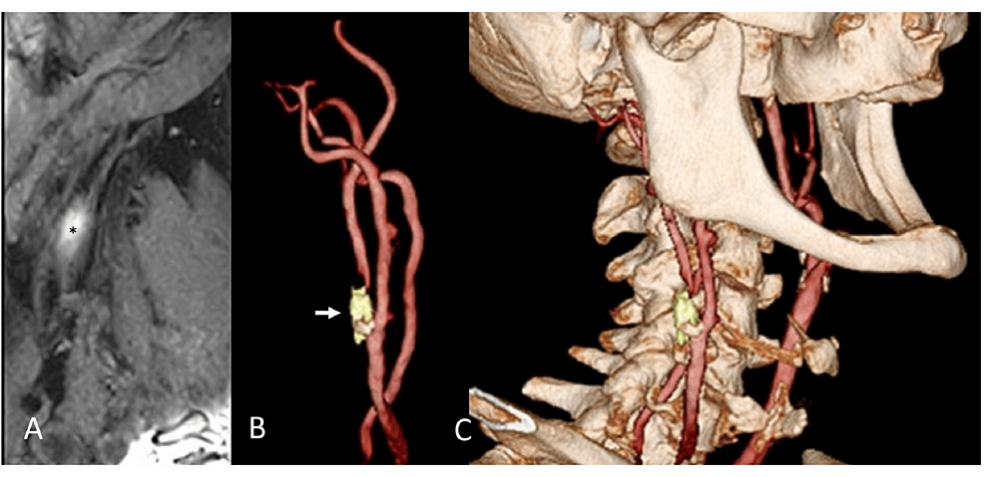
Preoperative fusion image of carotid endarterectomy (A) The reconstructed sagittal image of source images of T1-CUBE with fat suppression shows a T1 hyperintense signal, indicative of intraplaque hemorrhage (asterisk). (B) Carotid artery 3D time-of-flight magnetic resonance angiography (3D TOF-MRA), right internal carotid artery origin showing severe stenosis (arrow), overlaid with unstable plaque and calcified component. (C) Direct surgery was considered appropriate based on the characteristics of the carotid plaque and its anatomic location relative to the mandible.

MR angiography (MRA) revealed a short, severe stenosis at the beginning of the right ICA and no stenosis in the left ICA. The intracranial main arteries, including the vertebrobasilar artery (VBA) system, were well delineated. The A1 segment of the anterior cerebral artery (ACA) between the carotid bifurcation and the origin of the anterior communicating artery was nondominant on the right side, and the bilateral posterior communicating arteries (PcomAs) were of the adult type. Although the arterial-spin-labelling MRI is still limited in its use as a cerebral perfusion assessment, we had to use it to assess cerebral perfusion status in this case, suggesting that the CBF around the right insular cortex was decreased compared to the left side. MR plaque evaluation showed that the plaque consisted mainly of intraplaque hemorrhage with some calcification, suggesting that direct surgery was the most appropriate approach, considering the plaque's localization on fusion imaging (Figure 2) [8].

**Figure 3:**
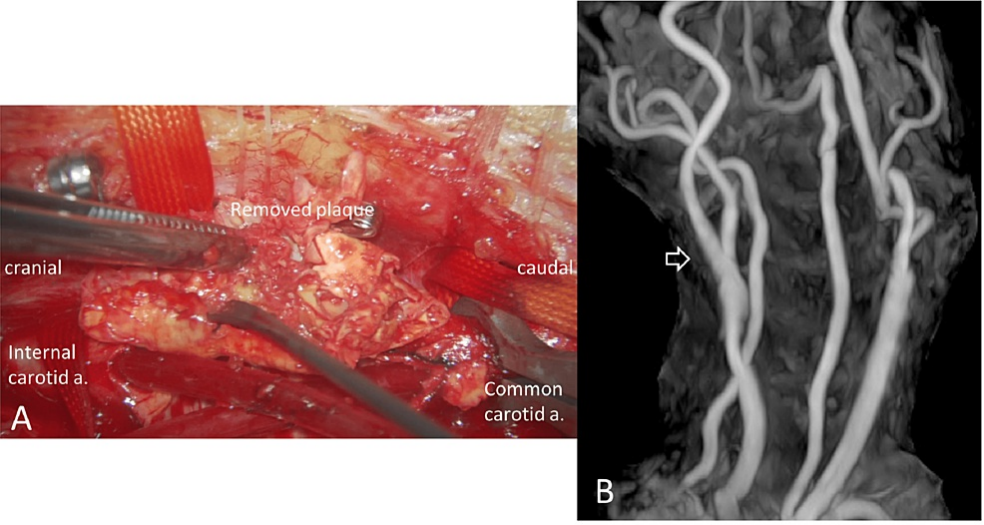
Intraoperative finding and postoperative magnetic resonance angiography (MRA) (A) Intraoperative finding showed an unstable plaque composed primarily of intraplaque hemorrhage, as noted in the preoperative MR plaque evaluation. (B) Postoperative reconstruction of the internal carotid artery was good.

Under general anesthesia, CEA was performed with an internal shunt while monitoring the motor-evoked potential (Figure 3). Carotid artery revascularization was well-achieved (Figure 3), and the patient had no postoperative ischemic or hemorrhagic complications or lower cranial nerve palsies, providing reassurance about the safety of the procedure.

Postoperatively, syncope attacks wholly resolved, even when her systolic BP fell below 110 mmHg. The patient was free of syncopal symptoms on clinical follow-up at one month and six months.

## Discussion

Syncope, a condition caused by a transient decrease in CBF to the diencephalon or mesencephalon, including the reticular formation and its ascending pathway, parts of the brain that control consciousness, is a complex condition. A decrease in systemic BP usually accompanies syncope, and the significant etiologies reported are vasovagal reflex (21.2%), cardiogenic (9.5%), orthostatic hypotension (9.4%), and unexplained in 36.6% of cases [9,10]. Vasodepression predominates over cardioinhibition as the primary hemodynamic mechanism of vasovagal reflex syncope in the elderly, mainly due to transient inhibition of the sympathetic nervous system [10]. Another form of reflex syncope, carotid sinus hypersensitivity, has been reported in a significant proportion of patients without a history of syncope or falls, and its definition as a disease remains controversial [10]. However, that has been reported to be associated with altered responsiveness of carotid sinus baroreceptors due to carotid stenosis or chronic denervation of the sternocleidomastoid muscle, and the possibility that CEA affected these in the present case cannot be excluded [11]. The presenting patient was referred to our hospital after a thorough examination by a cardiologist ruled out cardiogenic syncope. Orthostatic hypotension is often associated with syncope in the elderly. It is defined as a sustained drop in systolic BP of 20 mmHg or more or diastolic BP of 10 mmHg or more on the elevation of the head [12]. Before CEA, her syncope was accompanied by a drop in BP that met this definition. However, after CEA, she no longer experienced recurrent syncope even when her systolic BP fell below 110 mmHg. This fact suggested that orthostatic hypotension was not the primary cause of her repeated syncope and led us to believe that severe stenosis of the right ICA was intensely involved in this patient's syncope. While the association between VBA insufficiency and syncope is well-established, cases of syncope linked to carotid artery stenosis or occlusion are rare. Most cases are associated with bilateral severe stenosis or occlusion, and it was believed that syncope was triggered by decreased CBF across the bilateral global cerebral hemispheres due to dehydration or antihypertensive drugs [1,2]. A man in his 60s with severe bilateral ICA stenosis who developed syncope underwent carotid stenting of the left ICA only, and his syncope disappeared [2]. In the presented case, it is noteworthy that there was no significant stenosis in either the VBA system or the left ICA. However, severe stenosis was found only in the right unilateral ICA. This condition suggests that decreased CBF throughout the bilateral global cerebral hemispheres is highly unlikely unless the BP is extremely low. On the other hand, the left hemiparesis accompanied the syncope, and part of the right hemisphere was likely ischemic, and the area containing it was involved in the syncope.

Severe arrhythmias are often secondary to stroke, suggesting that the cerebral cortex plays a vital role in cardiac neuromodulation. Consistent associations with heart rate and heart rate variability have been reported in the anterior cingulate, amygdala, insular, and prefrontal cortex, but of great interest is the report that lateralization of the insular cortex is associated with autonomic cardiac responses [4,6,7]. Oppenheimer et al. [3] reported the results of stimulation of the insular cortex during temporal lobectomy in patients with a long history of epilepsy. They were the first to show that cardiovascular effects can be obtained in response to stimulation of the insular cortex in humans and that there is some degree of lateralization in the response. Although this report is based on a small number of five cases, they observed a trend toward activating the sympathetic nervous system by stimulating the right insular cortex. Meyer et al. [4] also reported that stroke patients in the right hemisphere, including the insular cortex, are more likely to develop cardio-autonomic dysfunction because of a marked pathological activation of the sympathetic nervous system. Royall et al. [5] reported that a significant proportion of healthy, undemented elderly patients without clinically significant cardiovascular disease, such as the present case, had asymmetric insular cortical CBF at rest, which is very interesting and may be related to autonomic-related morbidity and mortality in the elderly. In addition, in this report, the reduction in regional CBF in the right insular cortex was correlated with posture-induced BP reduction. It has been reported that left ventricular hypokinesis in a patient with herpes encephalitis resulted from decreased sympathetic nervous system activity due to the right-sided insular cortical lesion [13].

Furthermore, reduced right insular cortical volume in patients with neurogenic syncope was associated with more significant reductions in systolic and diastolic BP during the head-up tilt test [14]. The lateralization of the insular cortex concerning cardiovascular autonomic function remains to be elucidated. However, as we have discussed, many reports suggest that the left insular cortex is associated with vagal tone and the right insular cortex with sympathetic tone.

The insular cortex in the deep area of the Sylvian fissure receives blood supply mainly from perforating branches branching from the insular segment of the middle cerebral artery (MCA) [15]. In the present case, the right A1 is the nondominant side, so the bilateral ACA regions are mainly supplied with blood from the left ICA via the left ACA. Since the bilateral PcomAs are also of the adult type and the right MCA region receives its blood supply almost exclusively from the ICA, likely, the right insular cortex is somehow affected by the CBF decrease associated with the severe stenosis of the right ICA. The transient left hemiparesis concomitant with syncope attacks may also be a symptom reflecting ischemic loading to the right MCA region, including the insular cortex, which would support this. Even a decrease in BP that does not cause a decrease in CBF in the bilateral global cerebral hemispheres may cause a decrease in CBF in the right MCA region, including the insular cortex, which is solely dependent on the right ICA for blood supply due to severe stenosis of the right ICA, thus promoting hypotension and bradycardia due to a relative decrease in sympathetic effects. Although speculative, the above may be the pathologic mechanism of this patient's recurrent syncope attacks.

## Conclusions

We reported an older woman whose recurrent syncope attack wholly resolved after a CEA for severe stenosis of the right ICA. This woman had no significant stenosis of the left ICA or the VBA. Although the lateralization of the brain concerning the autonomic nervous system is gradually being elucidated, we speculated that the recurrent syncope attacks, in this case, were related to a relative decrease in sympathetic activity due to impaired CBF in the right cerebral hemisphere, particularly in the right insular cortex.
